# Quantitative Measurement Technique for Anodic Corrosion of BDD Advanced Oxidation Electrodes

**DOI:** 10.1021/acsmeasuresciau.3c00069

**Published:** 2024-02-23

**Authors:** Joshua J. Tully, Daniel Houghton, Ben G. Breeze, Timothy P. Mollart, Julie V. Macpherson

**Affiliations:** † Department of Chemistry, University of Warwick, Coventry CV4 7AL, U.K.; ‡ Centre for Doctoral Training in Diamond Science and Technology, University of Warwick, Coventry CV4 7AL, U.K.; § Spectroscopy Research Technology Platforms, University of Warwick, Coventry CV4 7AL, U.K.; ∥ Element Six (U.K.) Limited, Didcot OX11 0QR, U.K.

**Keywords:** boron-doped diamond, electrochemical advanced oxidation, corrosion, white light interferometry, acetic acid, corrosion rate measurement, electrode stability

## Abstract

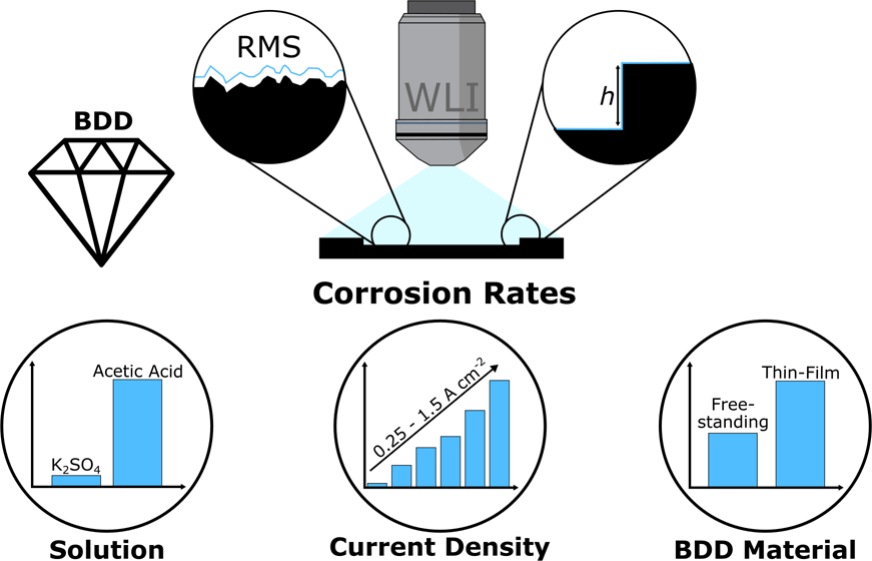

Electrochemical advanced oxidation (EAO) systems are of significant interest due to their ability to treat a wide range of organic contaminants in water. Boron doped diamond (BDD) electrodes have found considerable use in EAO. Despite their popularity, no laboratory scale method exists to quantify anodic corrosion of BDD electrodes under EAO conditions; all are qualitative using techniques such as scanning electron microscopy, electrochemistry, and spectroscopy. In this work, we present a new method which can be used to quantify average corrosion rates as a function of solution composition, current density, and BDD material properties over relatively short time periods. The method uses white light interferometry (WLI), in conjunction with BDD electrodes integrated into a 3D-printed flow cell, to measure three-dimensional changes in the surface structure due to corrosion over a 72 h period. It is equally applicable to both thin film and thicker, freestanding BDD. A further advantage of WLI is that it lends itself to large area measurements; data are collected herein for 1 cm diameter disk electrodes. Using WLI, corrosion rates as low as 1 nm h^–1^ can be measured. This enables unequivocal demonstration that organics in the EAO solution are not a prerequisite for BDD anodic corrosion. However, they do increase the corrosion rates. In particular, we quantify that addition of 1 M acetic acid to 0.5 M potassium sulfate results in the average corrosion rate increasing ∼60 times. In the same solution, microcrystalline thin film BDD is also found to corrode ∼twice as fast compared to freestanding polished BDD, attributed to the presence of increased sp^2^ carbon content. This methodology also represents an important step forward in the prediction of BDD electrode lifetimes for a wide range of EAO applications.

## Introduction

Research into electrochemical advanced oxidation (EAO) systems based on boron-doped diamond (BDD) electrodes is well advanced with significant literature supporting their use^1−5^ and commercial systems available. The BDD electrodes are typically used either in the freestanding form (removed from the growth substrate)^6^ or in a thin film form, where the BDD remains attached to a non-BDD growth substrate, for example, niobium, tungsten, and silicon.^7^ For all EAO applications, the BDD electrodes are currently polycrystalline, grown by chemical vapor deposition (CVD).^8^ Such BDD EAO systems have been shown to be capable of removing a wide range of organic-based chemical contaminants from aqueous systems, such as phenolic acids^9^ and chlorinated^10^ and perfluorinated organics.^11−13^ BDD-EAO configurations also have the advantage of a relatively small spatial footprint allowing them to be deployed in places where on-site wastewater treatment by traditional methods (such as incineration) would usually be impractical or impossible.^14^

The effectiveness of BDD-EAO systems results from the ability of the BDD electrode to electrochemically generate highly oxidizing hydroxyl radicals (^•^OH) in solution, from water oxidation, eq 1.
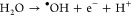
1

They, in turn, then react with organic species present in the solution (“indirect” route).^15−18^ This is due to ^•^OH being very weakly bound to the BDD surface when compared to other electrode materials,^17^ leading to the 1-electron water oxidation route dominating over 2-electron oxidation to hydrogen peroxide, 4-electron oxidation to oxygen, and 6-electron oxidation to ozone.^17,19^ Given the very high oxidizing power of ^•^OH, ^•^OH attack is thought to result in complete oxidation to CO_2_ and H_2_O for the majority of organic-based contaminants. “Direct” electrochemical oxidation or reduction of the organic species (and/or subsequent breakdown products) by the BDD electrode provides a complementary pathway for the degradative breakdown of organic species.

While BDD is well known for its chemical robustness and mechanical strength, it has been shown that at oxidative current densities >0.2 A cm^–2^^20^ or high applied anodic charge >100 A h cm^–2^_,_^21^ electrochemically induced corrosion of the surface is possible. Furthermore, the chemical identity of the organic pollutant present in the aqueous system has been demonstrated to play an important role in the corrosion process. For example, acetic acid at concentrations in the range 0.5–3 M^20,22^ causes significant corrosion of the surface when the BDD electrodes are operated at current densities of 0.5^22^ and 1.0 A cm^–2^.^20^ In contrast, sulfuric acid (1 M at 1.0 A cm^–2^),^22^ formic acid (2 M at 0.5 A cm^–2^),^20^ glucose (1 M at 0.5 A cm^–2^),^20^ perchloric acid (1 M at 0.5 A cm^–2^),^20^ and methanol (1 M at 0.5 A cm^–2^)^20^ are reported to show no observable change in the electrode surface.

To date, however, all corrosion studies concerning BDD have been qualitative in nature with information on corrosion being extracted from “before” and “after” measurements of the BDD surface (and not necessarily in the same area) using a range of techniques. These include (i) scanning electron microscopy (SEM),^20,23−25^ where the changes in grain morphology before and after corrosion are visualized; (ii) spectroscopy, in particular, Raman_,_^20,23−25^ glow discharge optical emission spectroscopy,^20^ energy-dispersive X-ray spectroscopy,^24^ X-ray diffraction spectroscopy,^24^ and X-ray photoelectron spectroscopy (XPS),^22^ where changes in the BDD surface are inferred from changes in the recorded spectra; and (iii) electrochemical measurements,^21,22,26^ such as measurement of the change in the solvent window and/or electrochemical double layer capacitance.

Such approaches have led to a far from complete and often contradictory picture. For example, Katsuki et al.^25^ found that the potential needed to maintain a fixed current density (0.1–2.0 A cm^–2^) at BDD electrodes in 10% sulfuric acid, increased as the boron dopant density decreased. They interpreted this as the BDD corrosion rate increasing in response to decreasing boron dopant levels. Kashiwada et al.^20^ showed that the (111) facets of BDD corroded faster than any other facets in 1 M acetic acid at 0.5 A m^–2^. In contrast, they attributed this result to the (111) facets containing the highest boron doping levels. In this study, it was also suggested that corrosion should not be possible unless organic species were present in the solution.^20^ This was supported by work from different authors that claimed no observation of corrosion (using SEM) for BDD electrodes operated at 1.0 A cm^–2^ for 256 h in 1 M H_2_SO_4_.^22^ In contrast, a complementary experiment, also from different authors, demonstrated corrosion (using SEM) under identical solution and current density conditions, and with a shorter experiment time.^27^ These inconsistencies in the experimental results could also be the reason for the different proposed mechanisms for anodic BDD corrosion. For example, Chaplain et al.^21^ suggest that ^•^OH is responsible for anodic corrosion while Kasiwada et al.^20^ claim that only radicals generated from organic species present in solution, via reaction with ^•^OH, contribute to corrosion.

Studies have also used different BDD film electrodes, which vary in terms of quality (amount of sp^2^ carbon content), thickness, and growth (support) substrates. Increased thickness typically results in larger crystallite sizes.^6^ To the best of our knowledge, all reported corrosion experiments have also been conducted under static conditions, with no flow to control the temperature, remove possible corrosion products, or deliver fresh reactant to the BDD surface, as would be typical in commercial systems.

To provide a quantitative understanding of the corrosion rates of BDD electrodes as a function of solution composition and current density, we present the first published laboratory scale method for the direct quantitative measurement of BDD anodic corrosion. The methodology is applicable to both thin film and freestanding polycrystalline BDD electrodes and operates over a reasonable time scale of 72 h. The set-up includes a custom-designed electrochemical flow cell, analogous to commercial systems, with flow being used to deliver reactive species, sweep away gases/soluble oxidation products, and deliver cooling. The set-up also prevents uncontrolled solution heating due to Joule heating and thus enables high current density operation. Post electrolysis, white light interferometry (WLI) is used to measure BDD recess depths on a pixel-by-pixel basis due to material corrosion. This approach also provides a method for the prediction of BDD electrode EAO lifetimes in a laboratory setting. It is complementary with the mass lost method, which is employed with larger commercial electrodes in the field, but the method reported herein importantly requires significantly shorter operational times (days as opposed to months/year).

## Experimental Section

### Chemicals and Solutions

All solutions were made using ultrapure water (Milli-Q, resistivity > 18.2 MΩ cm), and all chemicals were used as received with no further purification. Solutions were made using a mixture of potassium sulfate (K_2_SO_4_, Analysis grade, Sigma-Aldrich) and acetic acid (CH_3_COOH, 99%, Sigma-Aldrich).

### BDD Materials

**Figure 1:**
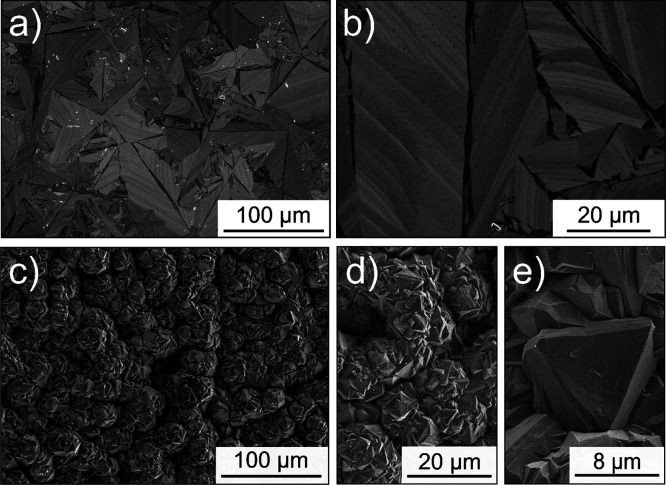
SEM images of the surface of the two different BDD electrodes used in this work. (a, b) Polished growth surface of EP grade freestanding polycrystalline BDD (Element Six) at high, a, and low magnification, b. (c–e) As-grown growth surface of thin film polycrystalline BDD attached to the growth substrate (DIAMCHEM, Condias) at (c) low, (d) medium, and (e) high (e) magnification. b and d are on the same scale to highlight the differences in the grain structure.

Electrochemical processing grade (commercially available Diafilm EP grade, Element Six Limited, Didcot, UK)^28^ polycrystalline BDD was used for the majority of this work. This material has been characterized in previous studies (electrode D in ref (29) is doped above the metallic threshold, average boron density of 3 × 10^20^ cm^–3^).^28^ The BDD is used in a freestanding form, that is, where it has been removed from the growth substrate. The surface of the BDD in direct contact with the underlying growth wafer is called the nucleation face, while the top surface is referred to as the growth face. The BDD was resin bond polished on the growth face, resulting in a typical roughness of ∼10 nm *S*_q_ (RMS, calculated by taking the square root of the arithmetic mean of squared heights), while the nucleation face was mechanically lapped to an *S*_q_ ∼ 100 nm. The final thickness of the BDD was ∼400 μm. Typical SEM images of the polished growth face (at low and high resolutions) are shown in Figure 1a,b.

The surface shows a distinctive “palm-tree” grain structure of long and thin grains. Note the striations in the grains, which are thought to be due to extended defects, such as twins, stacking faults, and dislocations.^30^ From the contrast seen in SEM, the narrow regions in between the larger grains, which are darker in color, likely indicate regions of higher boron uptake,^29^ although we cannot exclude the presence of sp^2^ carbon. A corresponding WLI image is presented in SI.1. The WLI image is on a much larger scale than the SEM images and shows height differences of ∼15 nm between high and low areas on the surface. Holes (∼20 nm deep) are also visible on the electrode surface.

The material was cut into 10 mm rounds using a 335 nm Nd:YAG 34 ns laser micromachining system (E-355H-3-ATHI-O), Oxford Lasers. After laser cutting, the electrodes were acid cleaned in a boiling mixture of concentrated sulfuric acid (H_2_SO_4_, >96%, Merk) and potassium nitrate (KNO_3_, 99.97%, Sigma-Aldrich) using a procedure described elsewhere.^31^ After cleaning, a bilayer ohmic metal contact (Ti 10 nm/Au 400 nm) was deposited onto the lapped (nucleation) surface using a Moorfield MiniLab sputter system. After deposition, this contact was annealed at 400 °C in air for 5 h.^31^

Thin film BDD was also used in these studies (Condias GmbH, Germany; DIACHEM) consisting of a ∼10 μm thick layer of BDD supported on an 8 mm diameter niobium substrate and was used as received. SEM and WLI of this BDD surface can be found in Figures 1c–e and SI.2, respectively. The as-grown surface of the DIACHEM electrode is much rougher (*S*_q_ value of 3.47 μm, SI.2) than the polished EP-grade BDD surface. The grain structure is also significantly different, with the thin film material consisting of cauliflower-like structures that are made up of multiple crystal facets, which is also evident from WLI (SI.2).

### Anodic Corrosion Experiments

**Figure 2:**
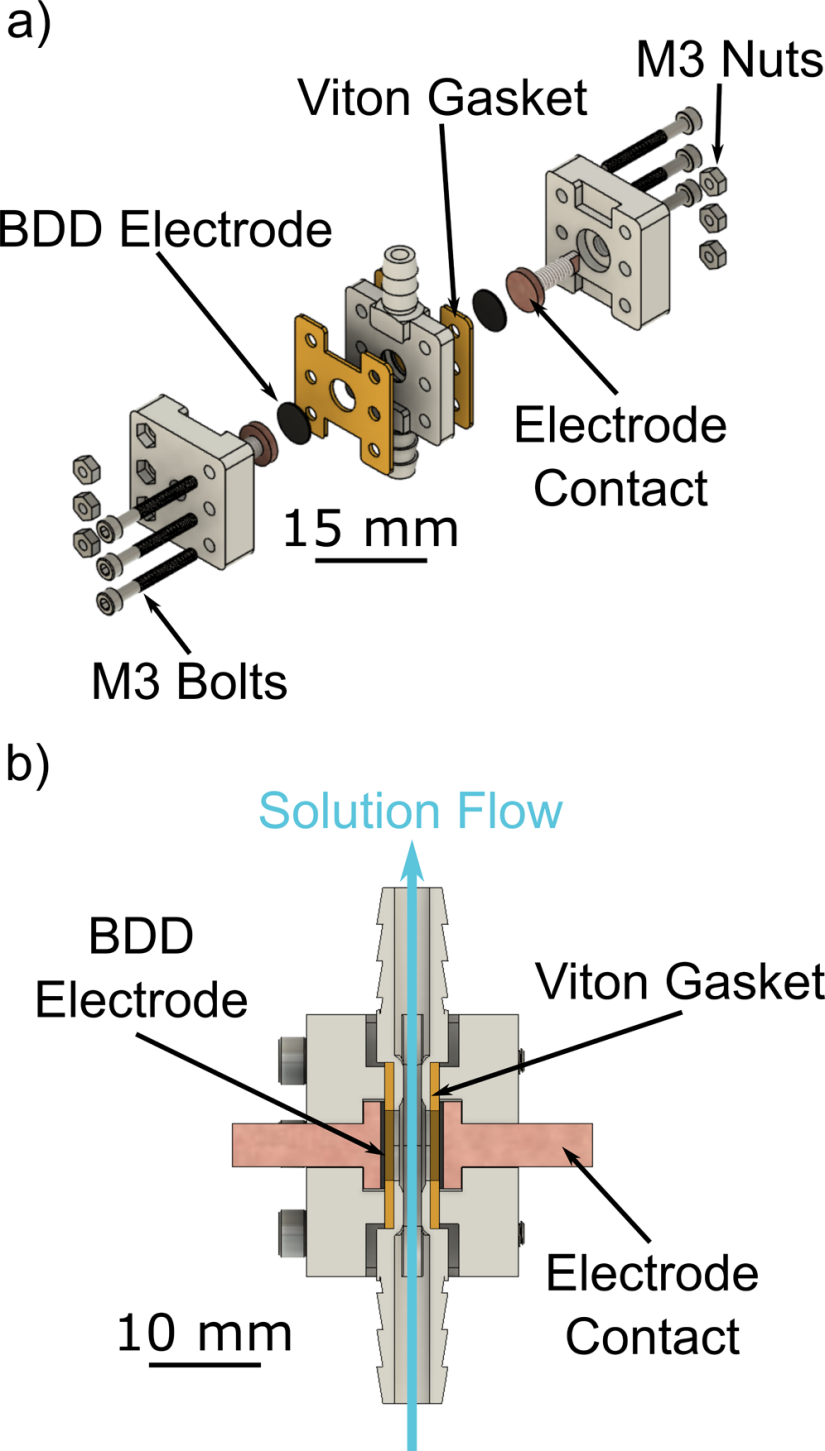
Render of the flow cell designed for the BDD anodic corrosion experiments. (a) Expanded view showing all the cell components. (b) Cross section through the assembled cell showing the flow path for solution.

To study and quantify anodic corrosion, a custom cell was specifically designed and fabricated (Figure 2) which contained two 10 mm diameter BDD electrodes (the anode and the cathode). When designing the cell, five important features were considered. First, the BDD electrodes had to be easily removable for cleaning and corrosion quantification. Second, the cell needed to isolate part of the BDD surface from the solution, in order to prevent corrosion and provide a baseline reference against which the corroded surface could be measured. Third, the cell had to be able to tolerate high currents, which necessitated the use of only metal electrical components (i.e., no conductive adhesives). Fourth, the cell was designed to accommodate a flow path. Flow is important in these experiments as it provides reactants and removes products from the electrode surface. It also serves to control the temperature, as the solution is circulated from a temperature-controlled reservoir into the cell. Finally, the electrode spacing was also designed to be as small as possible to minimize ohmic drop and increase the face velocity of the solution for a given flow rate.

The final cell comprised three stereolithography 3D-printed parts, two brass electrode contacts, two Viton gaskets, and hardware to secure the cell together. The 3D components were printed in high-temperature resin (FormLabs, Massachusetts, US) using a Form 3 (FormLabs, Massachusetts, US) stereolithography 3D printer. High-temperature resin was used to prevent deformation of the cell during experiments at elevated temperatures. The gaskets used to seal the cell were 1 mm thick Viton cut to size (Delta Rubber, UK). The two BDD electrodes were placed so that the Ti/Au contacts were in direct connection with the brass electrode contact, as shown in Figure 2a. This orientated the polished BDD growth face toward the solution flow channel, Figure 2b. Thin film BDD was also used in this configuration. BDD was chosen as the cathode material to prevent any solution contamination caused by cathodic corrosion of a metal electrode.^32^

The Viton gasket was used to seal the BDD electrodes such that an 8 mm diameter area (freestanding) or a 6 mm diameter area (thin film) was exposed to solution with a 6 mm separation between electrodes, through which the solution flows. The masked surface, which is not in contact with solution, provides a reference against which to measure surface loss due to anodic corrosion. The electrodes were connected to a variable DC power supply (EA-PS 9750-04, Elektro-Automatik GmbH, Germany) which enabled sufficient potential to be applied to maintain the desired current density for the duration of the experiment (72 h). The potential was allowed to vary to maintain the desired current density and fluctuated by ±2 V. Typically, the potentials at the end of the experiment were found to be slightly lower (1–2 V) than at the start. This is most likely due to the increased electrode area as a result of anodic corrosion.

In the majority of the experiments, a current density of 1.0 A cm^–2^ was employed. This current density is typical of those used for accelerated lifetime testing for EAO systems using thin film BDD electrodes^21^ and is also typical for the operation of commercial EAO systems utilizing freestanding BDD electrodes.^33^ Solution was passed through the cell, at a rate of 200 mL min^–1^, from a 1 L reservoir in a temperature-controlled water bath at 60 °C. This setup ensured that the temperature of the experiment remained constant and provided temperature conditions more akin to commercial BDD-EAO cells.

### White Light Interferometry

All WLI interferometry reported in this work was collected by using a Bruker Contour GT-K instrument (Bruker, Germany). Data was analyzed in Gwydion version 2.55. WLI was used to both quantify the recess depths of the anodically corroded surfaces and determine the surface roughness of the BDD disk pre- and post-anodic corrosion.

### Recess Depth Measurements

WLI images in the vertical scanning interferometry (VSI) mode were recorded once the BDD disk had been removed from the cell to quantify the recess depths of the corroded surface. To do this, the 5× objective and 0.55× zoom lenses were employed, giving a total magnification of 2.75× and a field of view (FOV) of 2.3 × 1.7 mm (the image *x, y* dimensions). Six of these 2.3 × 1.7 mm images were recorded across the diameter of the disk and then stitched together, with a 20% overlap in *x*, to give a final image 10 × 1.7 mm, which contains both the corroded and uncorroded periphery regions of the disk, as shown schematically in SI.3. Each image contains 1.3 megapixels, and in this configuration, each pixel is a 3.60 μm square.

Due to strain in the freestanding material, over the length of the cut disc, the BDD bows slightly inward or outward depending on where on the wafer the BDD round was cut from. SI.4a shows the WLI image of a BDD disc prior to corrosion. For this particular disk, the center of the BDD disk bows outward by ∼80 nm compared to the edge. To reduce the impact of the bow, the “remove polynomial background” tool in Gwydion was used. This tool automatically fits a polynomial of a specified order (between first and eleventh) and subtracts it from the data to best flatten the image. A second-order polynomial fit was chosen as this function best describes the bowed shape of the BDD. The result of this processing on the data in Figure SI.4a is shown in Figure SI.4b,c. This tool was also used to “bow correct” the WLI data of the corroded disks. Once corrected, the masked (uncorroded) regions were set to zero and the average depth of the entire corroded region was extracted using the Gwydion statistical quantities tool.

### Roughness Measurements

To measure the roughness (the *S*_q_ parameter) of the starting, uncorroded, and subsequently corroded BDD surface, an image was collected in VSI mode with a 5× objective and 1× zoom optic. This gave a total magnification of 5× and a FOV of 1.25 × 0.95 mm (image size). In this configuration, each image pixel is a 1.95 μm square. Images were recorded in the central region of the disk, as shown in SI.3 for pre- and post-corrosion experiments. From these images, the *S*_q_ value is extracted using the statistical properties tool in Gwyddion. As above, a second-order polynomial was also subtracted from these data before the roughness values were extracted.

### SEM Imaging

Field emission scanning electron microscopy (FE-SEM) was used to image the electrodes, using the in-lens detector on a Zeis Gemini FE-SEM 500 instrument operating at 4 kV.

## Results and Discussion

### Anodic Corrosion in Sulfate Electrolyte

WLI is a non-contact optical profilometry technique in which interference fringes caused by differences in the path length between a reference beam and a beam reflected from the sample are used to construct a 3D surface for the sample.^34−37^ WLI was preferred as a means to measure quantitative changes in the surface arising from anodic corrosion as opposed to atomic force microscopy (AFM) due to its ease of use, rapid data collection, mm-sized field of view, high *z* resolution (<0.01 nm), and large (10 mm) *z* scan range.

**Figure 3:**
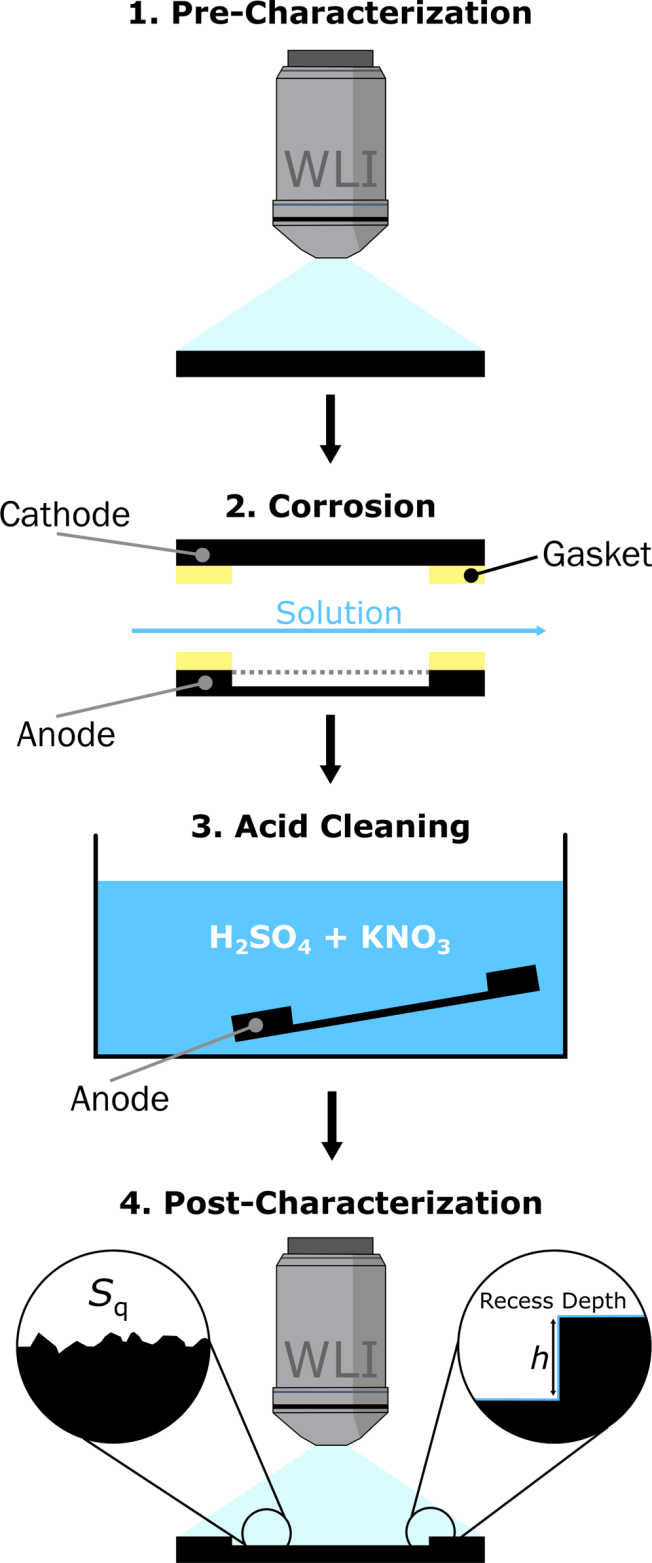
Schematic of the experimental procedure showing: (1) pre-characterization of the BDD electrode surfaces with WLI. (2) Corrosion of the solution-exposed region of the BDD anode. (3) Post-corrosion acid cleaning of the electrode. (4) WLI post-characterization of the corroded anode surface to determine recess depth and *S*_q_ roughness.

To validate the use of WLI as a measurement technique for quantifying anodic corrosion, an experiment was run for 72 h in a solution of 0.5 M K_2_SO_4_ at a current density of 1.0 A cm^–2^ (the applied potential varied between 13.2 to 14.8 V) using polished EP grade freestanding BDD. Figure 3 shows the experimental protocol for these experiments.

After electrolysis, both the BDD anode and cathode were acid cleaned (see Experimental), and the extent of corrosion was quantified using WLI. Note, acid cleaning is commonly used to clean diamond and BDD surfaces^38^ and is not expected to change the surface of the corroded electrode. For the cathode, WLI showed no appreciable change in the BDD electrode surface when comparing pre- and post-corrosion characterization data. This was verified for each new solution tested. An example of the post-corrosion WLI data on a BDD cathode is shown in SI.5 for the 0.5 M K_2_SO_4_ solution. One study in the literature demonstrates cathodic corrosion and film delamination of BDD (using AFM and SEM),^39^ but this was using thin film BDD (grown using hot filament CVD) under acidic conditions (0.5 M H_2_SO_4_) at >6000 C cm^–2^.

**Figure 4:**
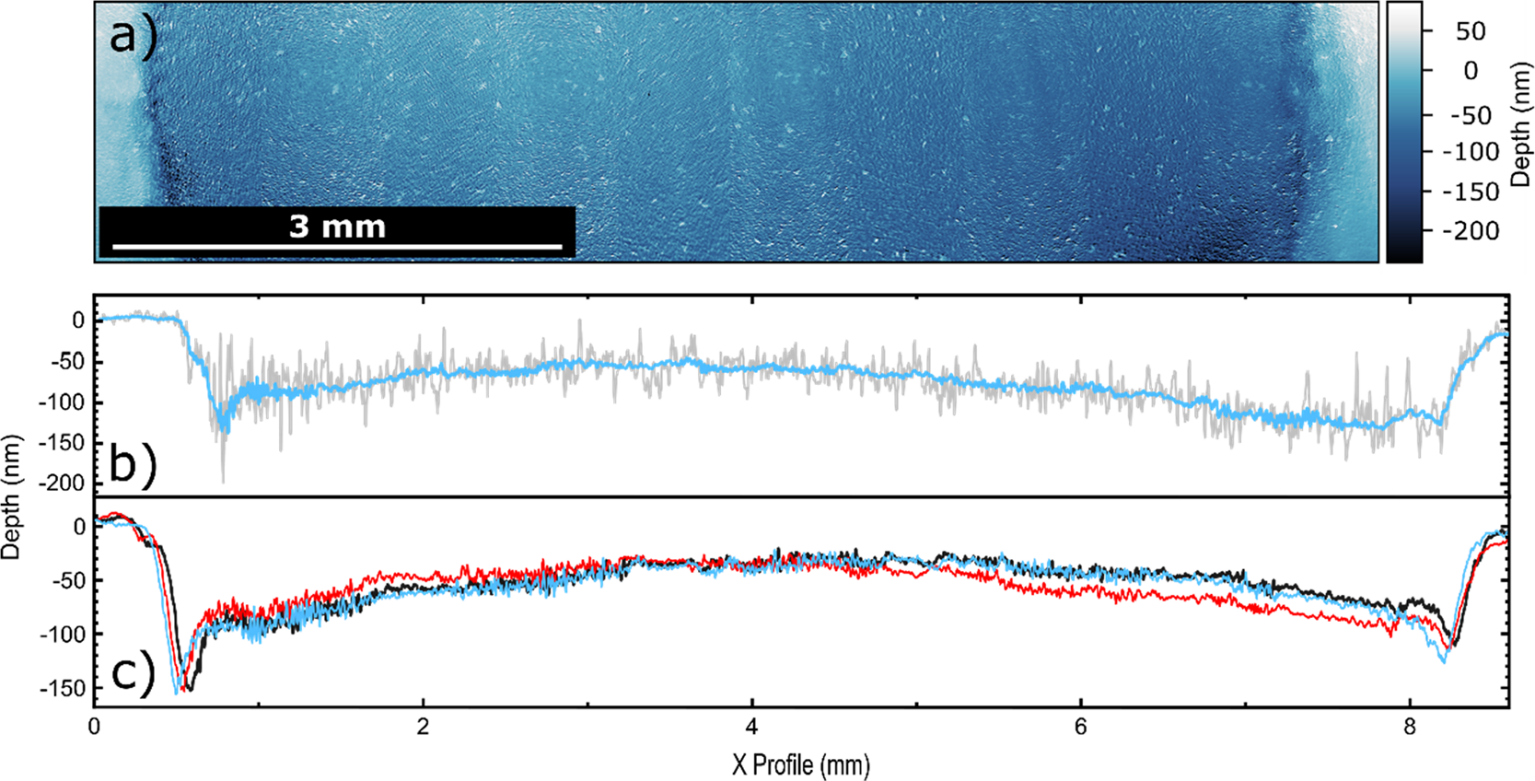
EP-BDD anode characterization after 72 h electrolysis at 1.0 A cm^–2^ in 0.5 M K_2_SO_4_. (a) WLI image of the surface, showing the smooth masked edge (light blue) and rougher corroded region (dark blue). (b) Line profile in *x* across the center of the WLI image showing the change in height for a 5-pixel (18 μm) averaged region (gray) and a 128-pixel (460 μm) averaged region (blue). (c) 128-pixel averaged line profiles for all three experiments tabulated in Table 1.

Figure 4a shows the WLI data recorded over the central rectangular region of the disk (edge-to-edge), which comprises six WLI images stitched together, as described in the experimental section and SI.3. The darker blue area is the corroded region of the 10 mm diameter disk, while the lighter blue area is the uncorroded, periphery region.

Figure 4b shows the line profile in *x* across the midpoint of the WLI image, showing the change in recess depth for a 5-pixel wide (18 μm) averaged region (gray) and a 128-pixel wide (460 μm) averaged region (blue). The 5-pixel wide profile gives a more indicative representation of the surface topography, whereas the 128-pixel wide profile (the maximum possible in Gwydion) averages out the roughness and makes it easier to visualize the overall shape of the corroded region. The change in recess depth due to anodic corrosion is clearly visible, as is the increase in surface roughness caused by anodic corrosion. The corroded region has a distinctive shape, being deeper at the edges and shallower in the middle, even after removal of the original bow in the BDD, via polynomial subtraction, as described *vide supra*.

The deeper corrosion profile at the edges is likely to be due to increased mass transport caused by turbulent solution flow around the gasket, with this having the least effect in the middle of the disk. The WLI image and the 5-pixel wide line profile across the center of the image are shown in SI.6 before removal of the polynomial background (Figure SI.6a,b respectively). Both show that the original bow on this sample curves the opposite way to the central corroded region, and therefore, the corrosion profile is reflective of the true morphology rather than the residual bow remaining after polynomial subtraction.

For quantification, the average depth of the corroded region (measured by WLI), obtained by summing the recess depths for all pixels and dividing by the number of pixels, in combination with the experiment time scale (72 h), is used to determine an average corrosion rate. The corroded region in Figure 4 is determined to be recessed 61 ± 30 nm compared with the masked (uncorroded) edge. An average value was taken rather than the deepest (140 nm) or shallowest (45 nm) pixel to give a more accurate average corrosion rate for the electrode. From this value, an average corrosion rate in nm h^–1^ was obtained = 0.85 ± 0.42 nm h^–1^. This result differs with conclusions made in previous work using thin film diamond, which suggested (via SEM and electrochemical measurements) that anodic corrosion of BDD was not possible unless organic species were present in the solution.^20^ In contrast, our work uses BDD starting surfaces of much lower roughness (polished freestanding BDD) where very small changes in 3D surface morphology, as a result of anodic corrosion, can be accurately quantified using WLI.

To examine the repeatability of the corrosion rate measurement in the 0.5 M K_2_SO_4_ solution, the experiment was repeated a further two times (*n* = 3). The average recession depths for all three experiments are shown in Table 1, and the corresponding midpoint line profiles (over 128 pixels) are displayed in Figure 4c. These three experiments demonstrate good repeatability, suggesting that the height variability of the corroded surface does not significantly impact the repeatability of the measurement of the average recession depths, and provides confidence in measuring the average corrosion rate by this method. The three-line profiles also show good agreement, suggesting that the shape of the corroded region profile is also consistent between experiments.

**Table 1:** Tabulation of the Corrosion Depth and Average Corrosion Rate of Three EP Grade Freestanding BDD Electrodes in 0.5 M K_2_SO_4_ at 1.0 A cm^–2^ for 72 h

experiment	average depth of corrosion (nm)	average corrosion rate (nm h^–1^)	*S*_q_ of central region (nm)
1	61 ± 30	0.85 ± 0.42	35.1
2	45 ± 21	0.62 ± 0.29	47.3
3	52 ± 24	0.72 ± 0.33	36.0

As a rule, this technique is appropriate for measuring a corroded region which is recessed from the starting surface by a value greater than the *S*_q_ value of the starting surface. This is to ensure that the recession due to corrosion can be reliably measured using WLI. Here, the initial average *S*_q_ of all three rounds is 6.0 ± 3.6 nm. The larger the corroded recess compared to the starting surface roughness, the more confidently the depth of the recess can be measured, with triplicate measurements improving confidence further. Note that to measure solutions which give rise to average corrosion rates that are too low to satisfy this requirement, the electrolysis time could be increased to achieve a corroded region recession of >*S*_q_.

For all three repeats, none of the BDD cathodes showed signs of corrosion using WLI and were reused for multiple experiments. While low, the average BDD anode corrosion rates measured for 0.5 M K_2_SO_4_ at 1.0 A cm^–2^ could still be significant for electrodes deployed in the field for long time scales of potentially years. While it is not possible to account for failure modes related to material weakening as the BDD gets thinner, if failure was due to electrode corrosion alone, a lifetime of ∼550,000 h in this solution can be estimated (assuming a starting electrode thickness of 400 μm and corrosion rate of 0.73 ± 0.10 nm h^–1^).^20^

From the data, it is also possible to quantify changes in *S*_q_ arising due to anodic corrosion (as detailed in the experimental section). The center of the corroded region had an *S*_q_ value of 40 ± 7 nm (*n* = 3, values shown in Table 1), SI.7. This is higher than the pre-corrosion polished surface which had an *S*_q_ value of 10 ± 4 nm (*n* = 3). A typical WLI image of the corroded region shows shallow holes (Figure SI.7 black circles >100 nm in diameter and ∼100 nm deep) and raised regions (red circles, approximately 20 μm in size and ∼50 nm high), the latter is consistent with grain size (Figure 1a,b). These are likely to be BDD grains that corrode slower than their neighbors leading to the observed grain relief versus the polished starting material.

We also note that the 1 mm wide masked edge of the anode had no appreciable increase in roughness after corrosion (*S*_q_ = 9.0 ± 1.9 nm after versus 10.0 ± 4.0 nm before), with the *S*_q_ post-corrosion value measured on three 1 mm squares of the uncorroded edge of the stitched images. This data confirms that the masked edge was successfully isolated from solution throughout the experiment and as such can be treated as a height reference for the corroded surface. While the *S*_q_ values for the central region of all three electrodes are reasonably consistent (Table 1), roughness only serves as an indirect measurement for the extent of corrosion on the electrodes and is less useful than the direct measurement of corrosion depth.

### Impact of Organic Content on Anodic Corrosion

**Figure 5:**
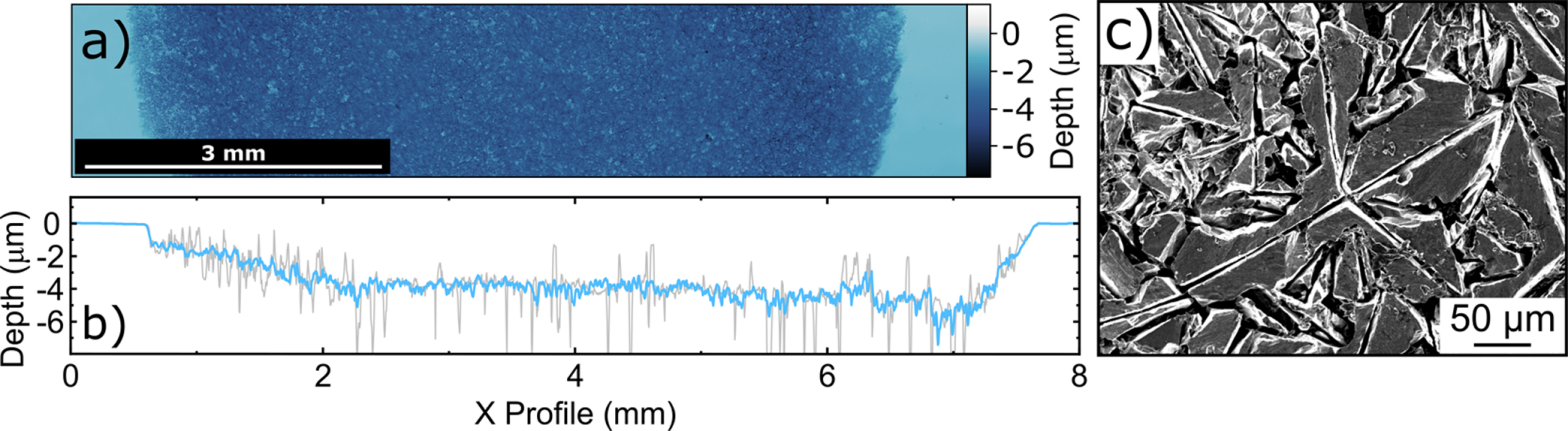
EP-BDD anode characterization after 72 h electrolysis at 1.0 A cm^–2^ in a solution of 1 M acetic acid and 0.5 M K_2_SO_4_. (a) WLI image of the surface, showing the smooth masked edge (light blue) and rough corroded region (dark blue). (b) Line profile in *x* across the center of the WLI image showing the change in height for a 5-pixel (18 μm) averaged region (gray) and a 128-pixel (460 μm) averaged region (blue). (c) SEM image of the corroded surface.

Acetic acid has been reported in the literature as a species that will cause significant anodic corrosion of BDD electrodes.^20,22,40^ This has been observed as a result of comparative SEM, XPS, and electrochemical measurements made on BDD anodes before and after electrolysis.^20,22^ The notable corrosion of the surface in these solutions is thought to be due to the production of ·CH_3_ radicals from reaction of acetic acid with electrochemically generated ^•^OH radicals to create CH_3_COO· which decomposes to form ·CH_3_.^20,41^ ·CH_3_ radicals are thought to be able to directly oxidize the BDD surface (leading to corrosion) via attack of surface C=O groups^20^ in the same way others believe ^•^OH interacts with the surface.^21^ To directly quantify the impact of acetic acid on the average anodic corrosion rate of BDD, a 1 M acetic acid +0.5 M K_2_SO_4_ solution was used for electrolysis, Figure 5. The 1 M concentration of acetic acid was chosen in line with the midrange of the previous literature.^20,22,40^Figure 5 shows post-electrolysis WLI and SEM characterization of the BDD anode.

The average recession depth of the corroded region of the BDD anode was 3.57 ± 1.43 μm below the masked edges; this gives an average corrosion rate of 50 ± 19 nm h^–1^, which is an increase of over 60 times compared to the average corrosion rate in 0.5 M K_2_SO_4_ alone (0.73 ± 0.10 nm h^–1^). This measurement quantifies previous qualitative work, which indicated that acetic acid causes significant corrosion of BDD anodes.^20,22^ The profile of the corroded region is markedly different from that seen in the K_2_SO_4_ corrosion experiment. The most etched regions are no longer those found around the edge of the corroded region. This is most likely due to the increased surface corrosion rate dominating over turbulent mass transport effects at the edges of the gasket.

The surface roughness of the central region of the corroded disk (1.25 × 1 mm, pixel size 1.95 μm) has increased from an *S*_q_ of 3.6 nm (data not shown) to 1.45 μm; over a 2 orders of magnitude increase. The corresponding WLI data (for the corroded disk) is shown in SI.8. As for the K_2_SO_4_ case, the WLI reveals holes and raised regions on the electrode surface. However, these are more significant, with larger holes around 50 μm in diameter and up to 10 μm deep and raised regions 50–100 μm in diameter and up to 4 μm in height. Some of these features can be seen as spikes in the 5-pixel averaged line profile (gray) in Figure 5b. SEM imaging, Figures 5c and SI.8, gives a higher resolution insight into the morphological changes associated with BDD anodic corrosion in acetic acid. First, there is a clear interface between the corroded and uncorroded regions, demonstrating that the gasket is successfully excluding the edge from solution. Second, it shows that corrosion is fastest at the narrow, most boron-doped regions, which sit between the larger grains. Although as noted earlier, these regions could also contain sp^2^ carbon. More detailed studies into the impact of boron dopant density and grain crystallography will be the subject of future work.

### Current Density

**Figure 6:**
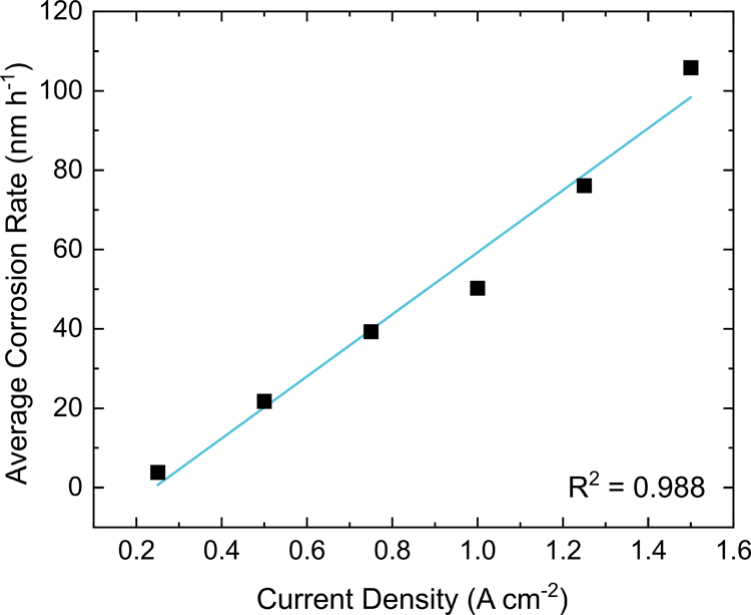
Average corrosion rates determined during 72 h electrolysis in a solution of 1 M acetic acid and 0.5 M K_2_SO_4_ at current densities from 0.25 to 1.50 A cm^–2^.

It has been previously suggested from qualitative observations that running EAO systems at lower current densities (<0.2 A cm^–2^) reduces the rate of anodic corrosion.^25^ To quantitatively explore the effect of current density on the anodic corrosion rate, a solution of 1 M acetic acid and 0.5 M K_2_SO_4_ was used. Experiments were conducted with current densities in the range 0.25–1.50 A cm^–2^ with both the average corrosion rates and surface roughness values measured. Current densities were set based on the starting geometric area of the electrodes (0.50 cm^2^). The average corrosion rates extracted from the data using the method discussed above are presented in Figure 6. For these experiments, 1 M acetic acid was added to 0.5 M K_2_SO_4_ in order to expedite corrosion, making it easier to measure differences in the average corrosion rate as a function of the current density using WLI.

Figure 6 shows that the average corrosion rate of the BDD electrode increases approximately linearly (*R*^2^ = 0.988) with an increase in current density, with a gradient of 78 ± 6 nm h^–1^ A^–1^ cm^2^. A table of the potentials required to pass each current density (at *t* = 0 s) is given in SI.9; note that these are not corrected for uncompensated resistance in the cells (Ohmic drop). The table shows that the higher the current density, the higher the starting voltage required. SEM, WLI, and line profiles for each corroded electrode can be seen in SI.10–SI.12, respectively. As current density increases, the average corrosion depth and roughness of the central corroded region also increases (SI.13).

Understanding the effect of current density is important for evaluating how appropriate a BDD-EAO system is for a particular application, as the current density will affect the rate of pollutant removal and therefore the size of electrodes required, which will in turn impact the start-up costs of the system. It also helps to contextualize accelerated lifetime testing typically used for thin film BDD electrodes, which employ higher current densities than would be used in normal operation to speed up electrode failure.^21^ While reducing the current density may seem the right approach for reducing anodic corrosion at the BDD electrode surface, the current passed through the electrode directly affects the amount of waterborne contaminants removed per unit time. As a result, reducing the current density will reduce the amount of contaminant the system can remove per unit time, unless larger area electrodes are used. The latter could be employed, but this will increase the cost of the electrodes and the space required for the cell.

The SEM images in SI.10 also qualitatively support a direct relationship between increasing current density and the rate of corrosion. At all current densities, the more highly doped regions between the large grains appear to have corroded preferentially. The WLI images (SI.11) of the central corroded region also demonstrate that corrosion occurs more favorably in the regions around the larger grains, which become darker, i.e. more recessed with increasing current density. The grain structure of the surface is also revealed more as the current density is increased, again as a result of the higher doped regions around the larger lower doped grains corroding preferentially.

Comparing the WLI line profiles for all of the current densities investigated (SI.12) reveals larger heterogeneities in the corrosion rate across the width of the corrosion region as the current density is increased. This can be seen most clearly for the 1.25 and 1.50 A cm^–2^ current densities, where one edge of the corroded region is deeper than the other. The origin of this asymmetry is currently unclear but will be the subject of further investigation. For high corrosion rate solutions, it may be beneficial to reduce the electrolysis time to minimize this asymmetry in the corrosion profile, when determining average corrosion rates in nm h^–1^.

### Thin Film Boron-Doped Diamond

**Figure 7:**
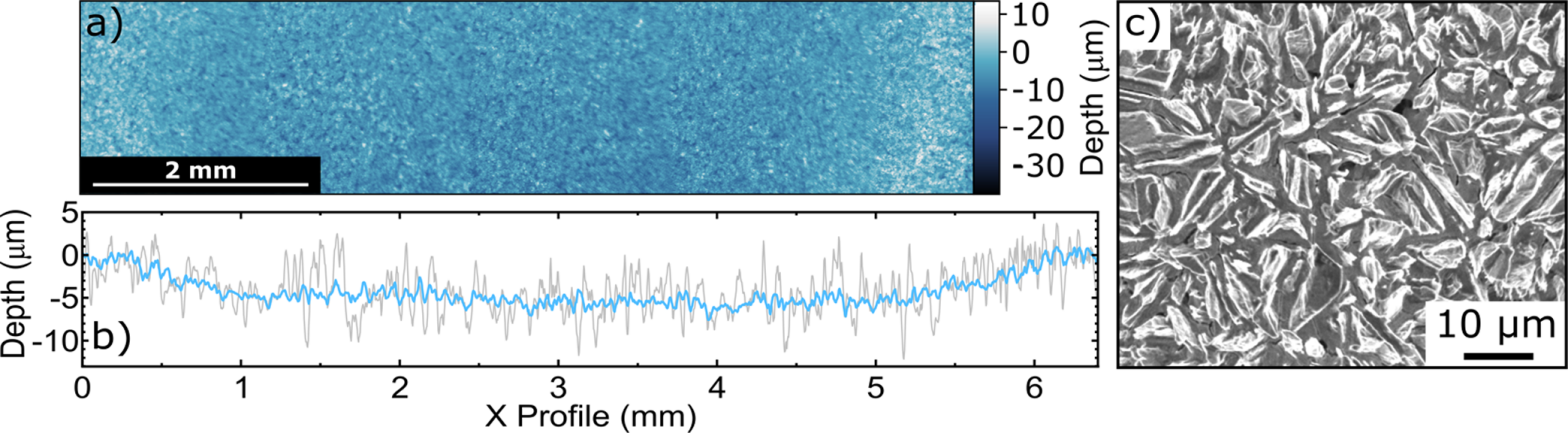
Thin film BDD anode characterization after a 48 h electrolysis at 1.0 A cm^–2^ in a solution of 1 M acetic acid and 0.5 M K_2_SO_4_. (a) WLI image of the surface, showing the higher masked edge and lower corroded region. (b) Line profile in *x* across the center of the WLI image showing the change in height for a 5-pixel (18 μm) averaged region (gray) and a 128-pixel (460 μm) averaged region (blue). (c) SEM image of the corroded surface.

Thin film BDD is widely used as the electrode material for EAO systems in conjunction with large surface areas and lower current densities. For accelerated lifetime testing of these systems, current densities of 1.0 A cm^–2^ are commonly used.^21^ As it is very challenging to reduce the starting surface roughness of thin film BDD electrodes by polishing, these electrodes were used in the as-grown state, which is akin to how they would be used in practical applications. The starting surfaces are significantly rougher (*S*_q_ = 3.47 μm) than the freestanding polished electrodes used above and as such limit the minimum recession of the corroded region that can be confidently measured using WLI to values >*S*_q_. The thickness of the thin film BDD layer is 10 μm. Given the much thinner BDD layer, a 48 h electrolysis time in a solution containing 1.0 M acetic acid and 0.5 M K_2_SO_4_ was employed instead of 72 h to prevent breakthrough of the BDD layer to the underlying niobium support. Figure 7 shows the post-corrosion WLI and SEM characterization of the anode surface.

The corroded area of this electrode was determined to be recessed by 4.89 ± 2.81 μm compared to the masked (uncorroded) area. This gives a corrosion rate of 101 ± 58 nm h ^–1^ for the 48 h experiment, which is over two times faster than that seen for the EP grade freestanding BDD. WLI and SEM characterization of these surfaces can be seen in SI.14. Interestingly, here, the roughness of the corroded surface of these electrodes is not significantly different from the starting surface; the roughness decreases slightly to 3.33 μm from the starting *S*_q_ of 3.47 μm measured for this electrode. The SEM images of the corroded surface reveal a grain structure that looks very different from that of the pristine material, with the round structures originally present (Figure 1c–e) now absent and replaced with small, narrow grains. It is interesting to note that the thin film BDD sample showed no evidence of a bow before or after corrosion likely due to it being supported on the thick niobium growth substrate. This work demonstrates the applicability of this measurement technique to quantifying corrosion rates in thin film BDD under accelerated lifetime test conditions.

Although a more detailed discussion of the basis for the higher corrosion rates seen with this material are beyond the scope of this publication, it may be due to the increased proportion of sp^2^ carbon present in this material.^21^ It can be seen from the SEM images in Figure 1 that this BDD has much smaller grains and therefore will contain a larger proportion of grain boundaries than the thicker freestanding material. The sp^2^ carbon content can also be assessed by using Raman spectroscopy (SI.15) prior to anodic corrosion. While it is clear from the sp^2^ carbon maps that both thin film and EP grade BDD have detectible levels of sp^2^ carbon, the thin film BDD Raman data shows that a larger proportion of the surface has a sp^2^ carbon signature.

## Conclusions

We present for the first time a laboratory scale method that enables the direct measurement and quantification of corrosion rate (in nm h^–1^) on BDD electrodes operating under EAO conditions, under flow, and constant elevated temperature. This methodology is demonstrated to be applicable to both thick freestanding and thin film (attached to the growth substrate) BDD anodes. The method uses WLI to directly quantify, on a pixel-by-pixel basis, the recession depth of the BDD surface, compared to the protected uncorroded surface, in response to an applied current density and measurement time. Using WLI, all the electrode surfaces exposed to electrolyte, in the cell, can be interrogated. This contrasts with qualitative techniques, such as SEM and Raman, which interrogate only small regions of the surface. Average corrosion rates as low as ∼1 nm h^–1^ were able to be measured using this methodology (for freestanding BDD with polished growth faces). Importantly, the ability to access such low corrosion rates enabled unequivocal demonstration and quantification of BDD EAO corrosion in electrolytes which do not contain organics; organics were previously thought to be essential in promoting BDD anodic corrosion under EAO conditions. From measurement of the corrosion rate in a laboratory setting over a period of several days, we suggest it will be possible to predict BDD electrode EAO lifetimes when deployed in the field over signficantly longer times.

Experiments carried out in 1 M acetic acid (an organic) and 0.5 M K_2_SO_4_ enabled quantification of a ∼60 times increase in the average corrosion rate compared to using 0.5 M K_2_SO_4_ alone, highlighting the role acetic acid plays in accelerating corrosion rates. In 1 M acetic acid (an organic) and 0.5 M K_2_SO_4_, a quantitative linear increase in the corrosion rate with current density was also demonstrated (specifically, an increase in the corrosion rate of 78 ± 6 nm h^–1^ for every 1.0 A cm^–2^ increase in current density). In the same solution, thin film electrodes showed a ∼2-fold increase in the corrosion rate compared to freestanding BDD anodes, a result attributed to the higher sp^2^ carbon content in the thin film material. This work highlights how important the composition of the effluent (solution matrix) and material properties of the BDD electrode are in determining the electrode lifetime. Future work will use this methodology to investigate further how solution and operational conditions impact corrosion rates as well as investigate the early stages of BDD corrosion using much shorter electrolysis times.

## Supporting Information Available

WLI of EP grade BDD starting surfaces; WLI of thin film-BDD starting surface; schematic of WLI measurements; WLI of bow in BDD; post-electrolysis characterization of the cathode; electrode profile before polynomial subtraction; WLI of the central region of EP grade BDD after electrolysis in K_2_SO_4_; post-electrolysis characterization of acetic acid corrosion; tabulations of potentials for current density experiments; SEM characterization for current density studies; central region WLI for current density studies; WLI profiles for current density studies; plot of corroded region roughness versus corrosion depth; WLI and SEM of the corroded Condias electrode; and Raman map of EP grade BDD and thin film BDD (PDF)
