# Supplementary material for: Quantitative Measurement Technique for Anodic Corrosion of BDD Advanced Oxidation Electrodes
Source: ACS Meas Sci Au. 2024 Feb 23;4(3):267–76. doi: 10.1021/acsmeasuresciau.3c00069 (PMC11191721; doi:10.1021/acsmeasuresciau.3c00069)
Supplement: Supplementary file 1 — tg3c00069_si_001.pdf [file tg3c00069_si_001.pdf]

Supporting Information

A Quantitative Measurement Technique for Anodic Corrosion of BDD Advanced Oxidation Electrodes

Joshua J. Tully,[1]* Daniel Houghton,[1,2] Ben G. Breeze,[3] Timothy P. Mollart,[4] Julie V. Macpherson[1]*

[1]Department of Chemistry, University of Warwick, Coventry, CV4 7AL, UK

[2]Centre for Doctoral Training in Diamond Science and Technology, University of Warwick, Coventry, CV4 7AL, UK

[3]Research Technology Platforms, University of Warwick, Coventry, CV4 7AL, UK

[4]Element Six (UK) Limited, Didcot, UK

*Corresponding Authors: Joshua.Tully@warwick.ac.uk; j.macpherson@warwick.ac.uk

SI.1: WLI of EP grade BDD Starting Surfaces

SI.2: WLI of thin film BDD Starting Surface

SI.3 Schematic of WLI Measurements

SI.4: WLI of Bow in BDD

SI.5: Post Electrolysis Characterization of the Cathode

SI.6: Electrode Profile before Polynomial Subtraction

SI.7: WLI of the Central Region of EP grade BDD after Electrolysis in K2SO4

SI.8: Post-Electrolysis Characterisation of Acetic Acid Corrosion

SI.9: Tabulations of Potentials for Current Density Experiments

SI.10: SEM Characterisation for Current Density Studies

SI.11 Central Region WLI for Current Density Studies

SI.12: WLI Profiles for Current Density Studies

SI.13: Plot of Corroded Region Roughness versus Corrosion Depth

SI.14: WLI and SEM of Corroded Condias Electrode

SI.15: Raman Map of EP grade BDD and thin-film BDD

SI.1: WLI of EP grade BDD Starting Surfaces

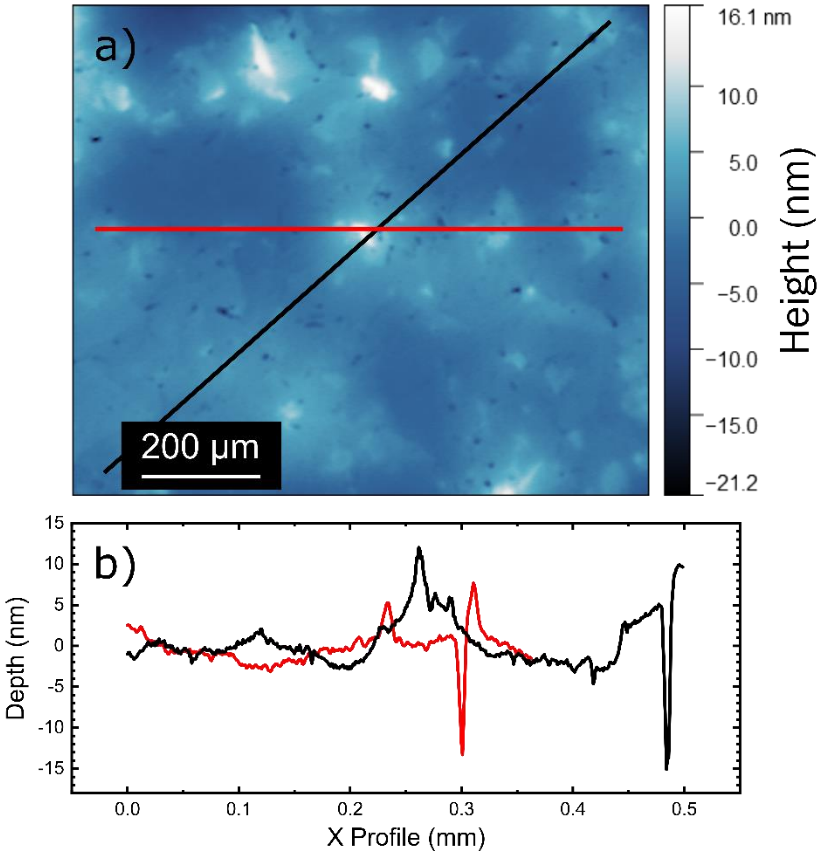

Figure SI.1: a) WLI image of a representative area of the EP-BDD polished surface prior to corrosion. b) Line profiles showing the height variation along the lines in a.

This image gives a root mean surface roughness, S_q, value of 7.4 nm. This value is determined from a single image (1.25 × 0.95 mm). Two other areas on the same sample were also measured (not shown) to give n = 3, the average S_q value for these three areas is 10 ± 4 nm.

SI.2: WLI of the thin film BDD Starting Surface

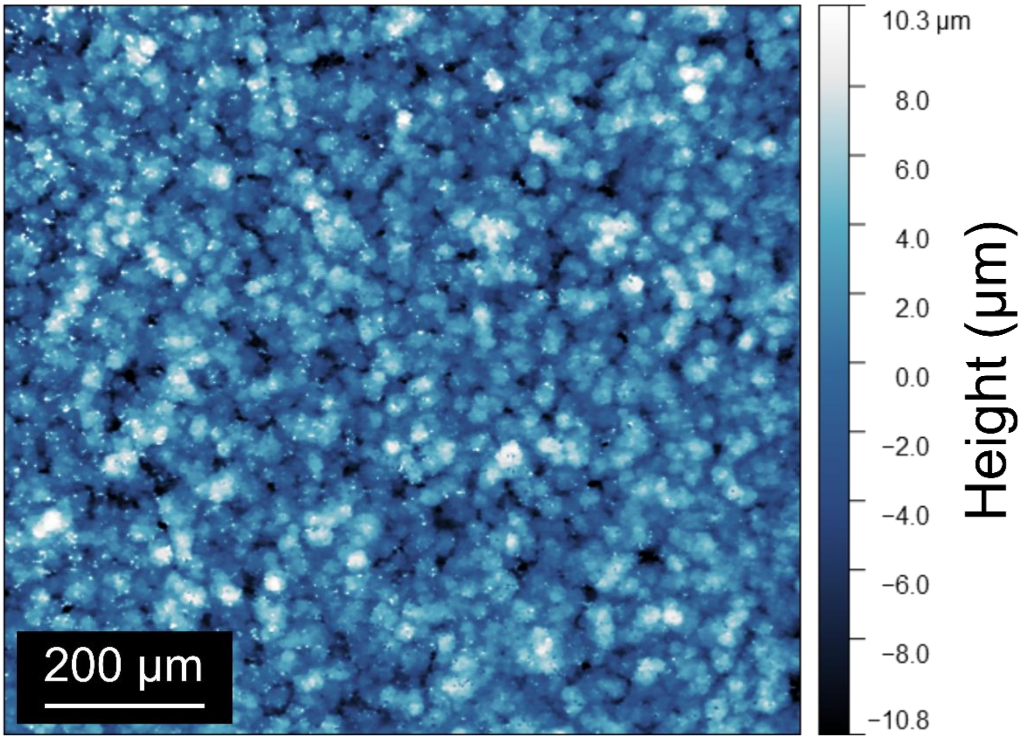

Figure SI.2: WLI image of a representative area of the thin film-BDD starting surface.

This image gives an S_q value of 3.47 µm, which was obtained in the central region of the electrode.

SI.3: Schematic of WLI Measurements

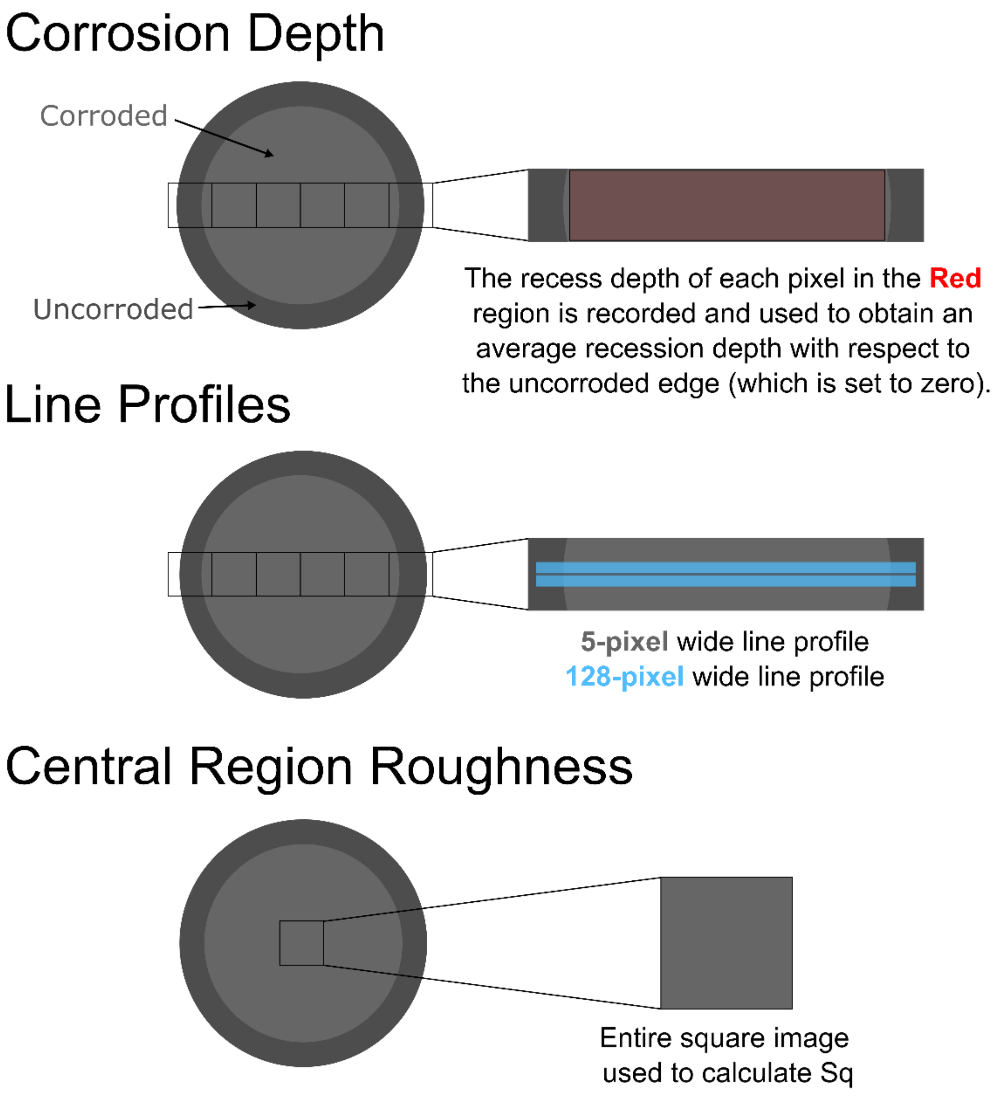

Figure SI.3: Schematic representation of how and where corrosion recession depth and surface roughness measurements are taken.

SI.4: WLI image showing bow in the BDD surface

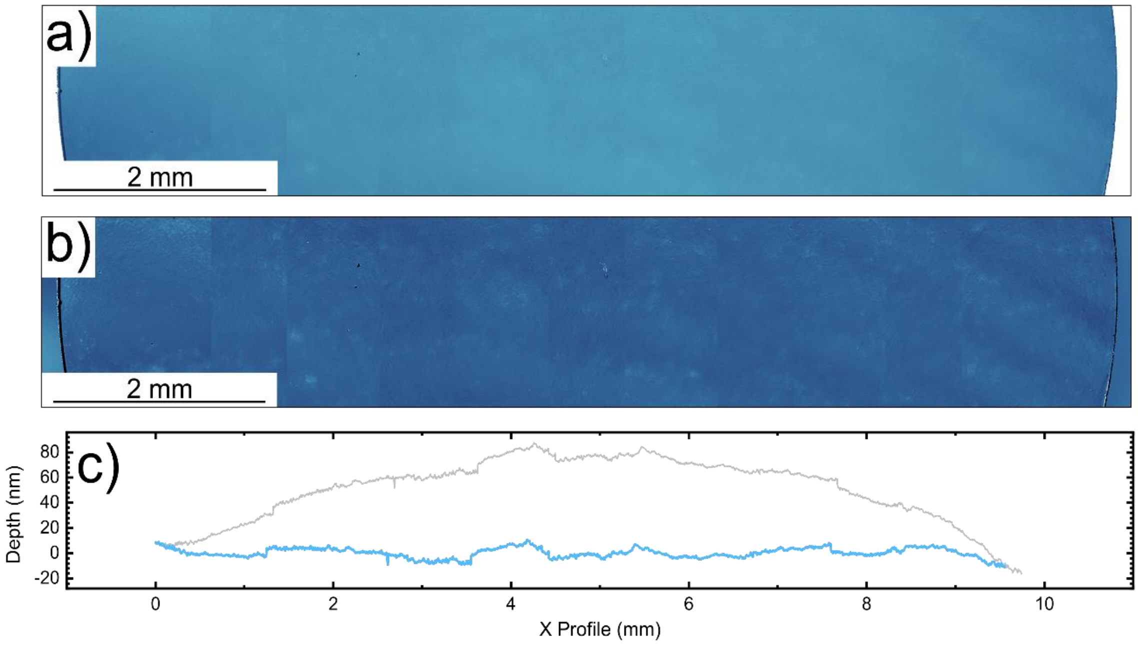

Figure SI.4: Demonstration of the bow present in an uncorroded 10 mm round of freestanding BDD. a) WLI image before polynomial subtraction. b) WLI image after polynomial subtraction. c) Line profiles for before (grey) and after (blue) polynomial subtraction. Note the slight discontinuities due to image stitching.

SI.5: Post-electrolysis Characterization of the BDD Cathode

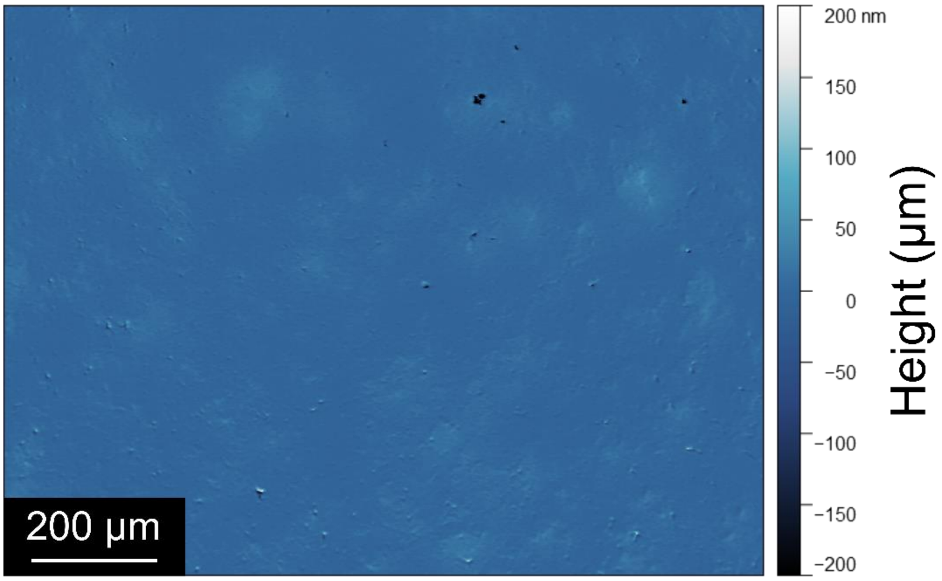

Figure SI.5: WLI characterisation of the cathode after 72 hrs of electrolysis in 0.5 M K_2SO_4 solution at 1.0 A cm^{-2}.

This image gives an S_q value of 8.1 nm. Three areas of the cathode were measured in total to give an S_q of 9.5 ± 0.5 nm. No significant difference is observed in roughness when compared to the electrode before electrolysis, which had an S_q of 10.6 ± 3.2 nm.

SI.6: Electrode Profile before Polynomial Subtraction

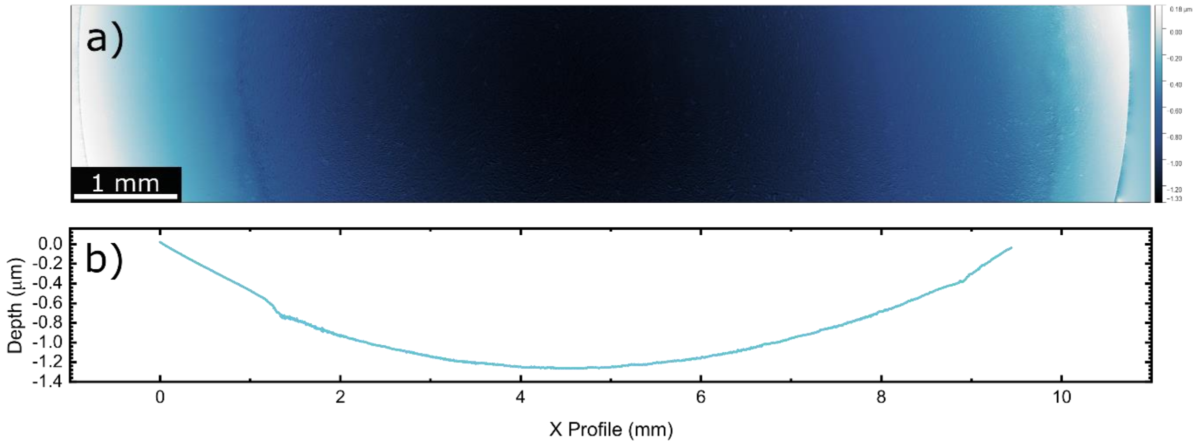

Figure SI.6: a) WLI image of the electrode from Figure 4a and 4b before polynomial background subtraction. b) 5-pixel wide line profile across a, showing the surface height changes.

SI.7: WLI of the Central Region of EP grade BDD after Electrolysis in K_2SO_4

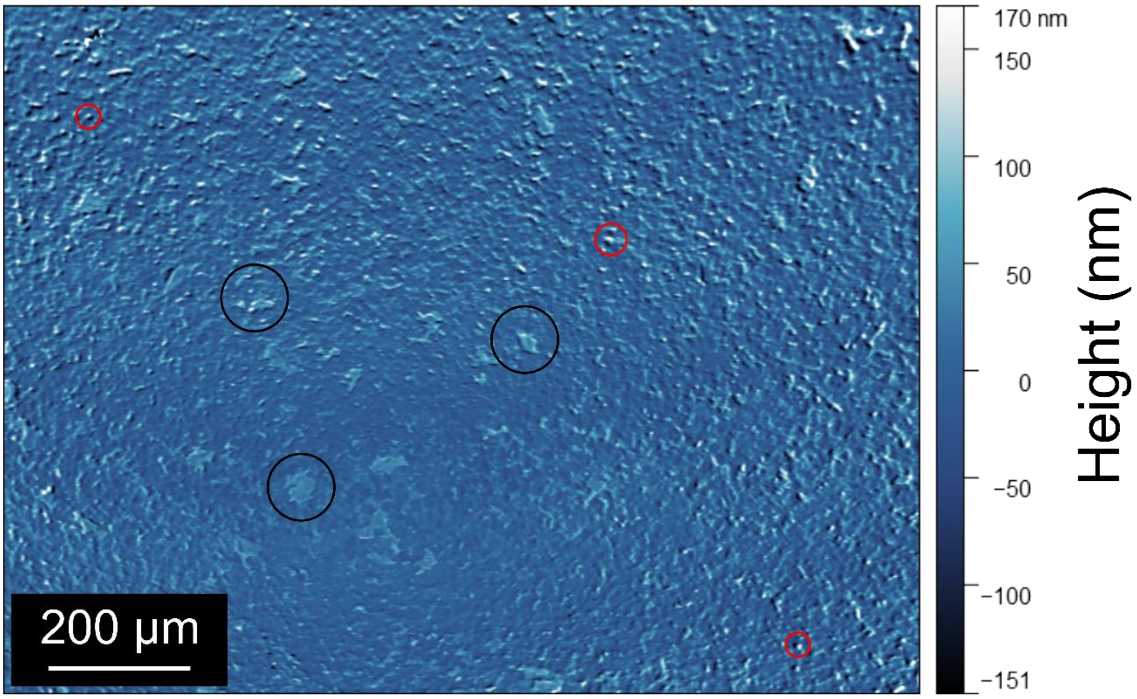

Figure SI.7: WLI image of the centre of an EP-BDD anode after 72 hrs electrolysis in 0.5 M K_2SO_4 at 1.0 A cm^{-2}. Black circles are used to denote some areas raised above the surface. Red circles are used to denote areas where there are holes in the surface.

This WLI image has an S_q value of 35.1 nm.

SI.8: Post-Electrolysis Characterisation of Acetic Acid Corrosion

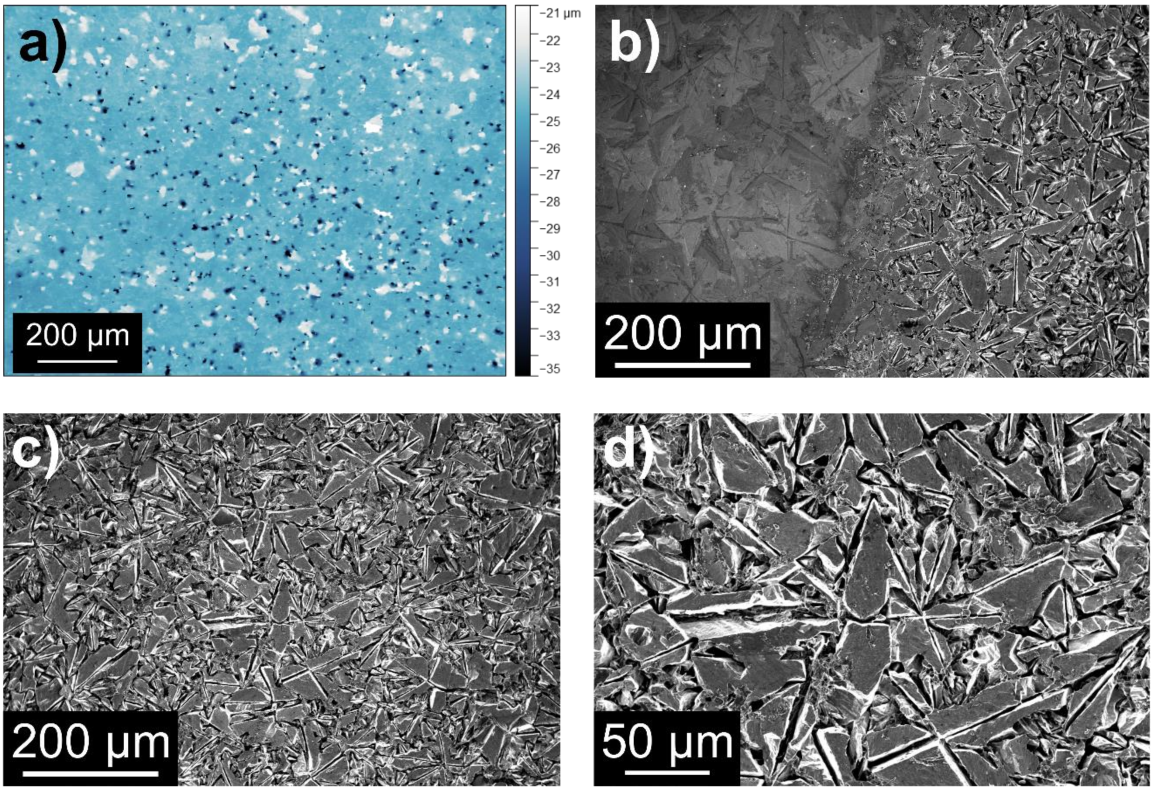

Figure ESI.8: Post-electrolysis characterisation of the BDD anode after 72 hrs electrolysis in 1 M acetic acid in 0.5 M K_2SO_4. a) WLI image of the centre of the corroded region showing the grain relief. In this WLI image, brighter regions are regions which stick up from the electrode surface, and darker regions are holes in the electrode surface. b) SEM image showing the clear interface between corroded and protected regions. c) 200× SEM image of the centre of the corroded region. d) 500× SEM image of the same region as c. All SEM images collected with a 4 kV acceleration voltage.

SI.9: Tabulations of Potentials for Current Density Experiments

Table S1: Tabulation of Currents and Potentials for Current Density Experiments

Current Density (A cm^{-2})	Current (A)	Starting Potential (V)
0.25	0.13	7.0
0.5	0.25	8.0
0.75	0.38	9.2
1	0.50	10.4
1.25	0.63	12.4
1.5	0.75	14.0

SI.10: SEM Characterisation for Current Density Studies

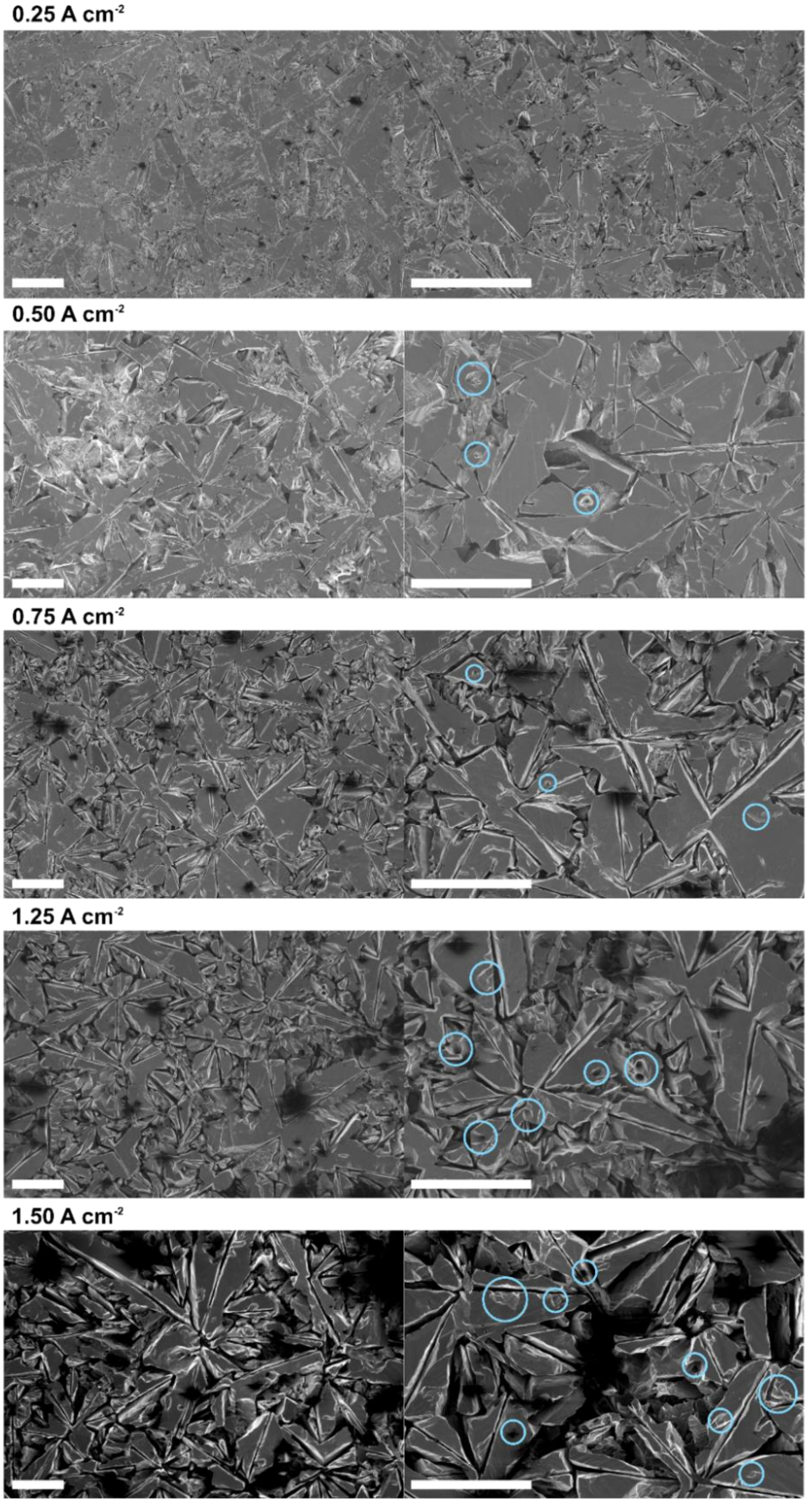

Figure SI.10: SEM images at 500× and 1000× showing the centre of the corroded region after 72 hrs of electrolysis in 1.0 M acetic acid in 0.5 M K_2SO_4 at each current density (labelled above the images). Scale bars on all images represent 50 µm. Blue circles correspond to regions where etching is occurring on-grain or along crystal directions.

SI.11 Central Region WLI for Current Density Studies

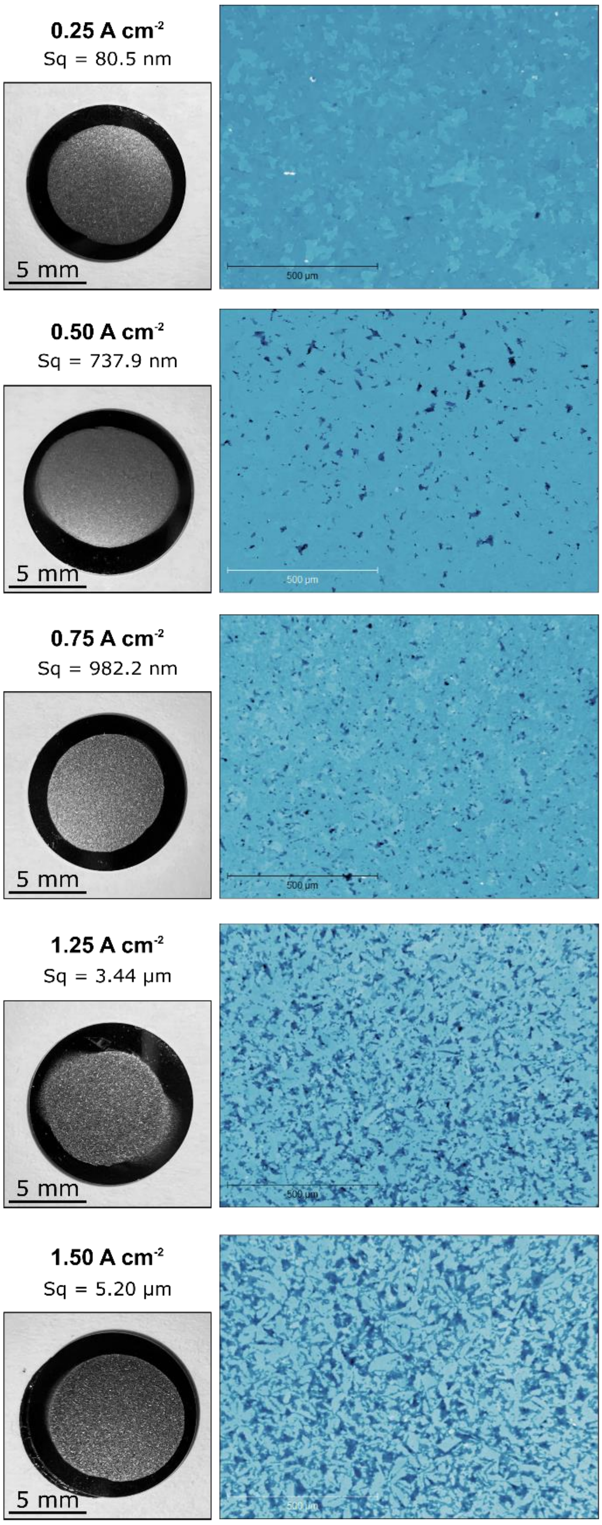

Figure SI.11: Left, photographs of the anode after electrolysis at each current density, taken with a camera phone. Right, WLI images of the central region of electrodes corroded at different current densities.

SI.12: WLI Profiles for Current Density Studies

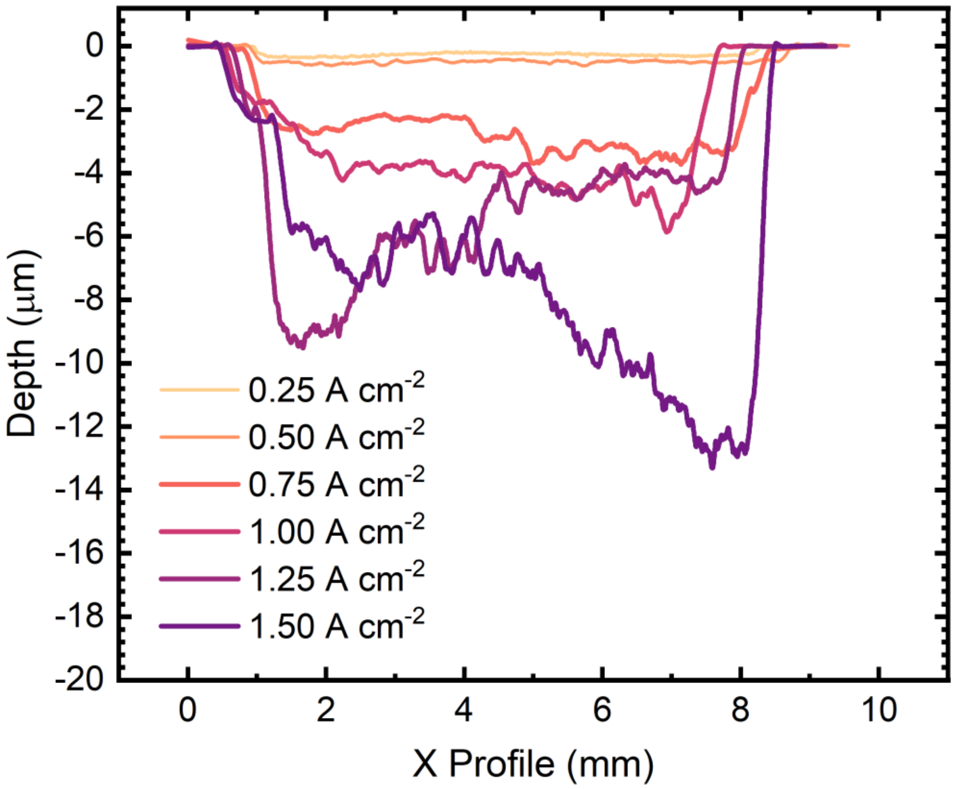

Figure SI.12: 128-pixel wide line profiles across BDD anodes electrolysed in 1 M acetic acid in 0.5 M K_2SO_4.

SI.13: Plot of Corroded Region Roughness versus Average Corrosion Depth

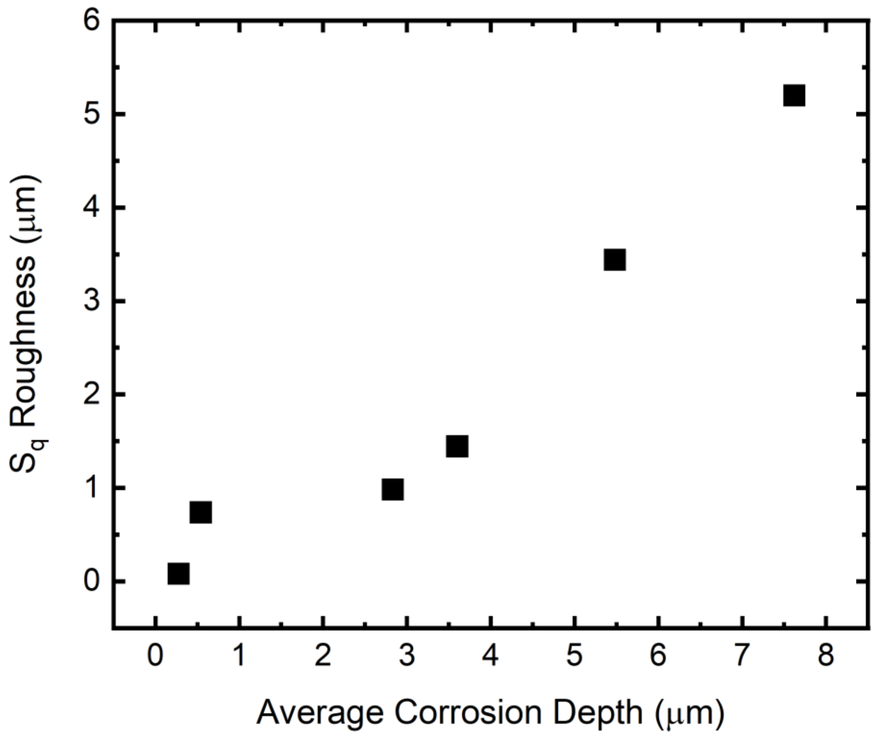

Figure SI.13: Plot of corrosion depth vs S_q roughness from WLI of the central region (black points).

SI.14: WLI and SEM of Corroded Thin Film BDD Electrode

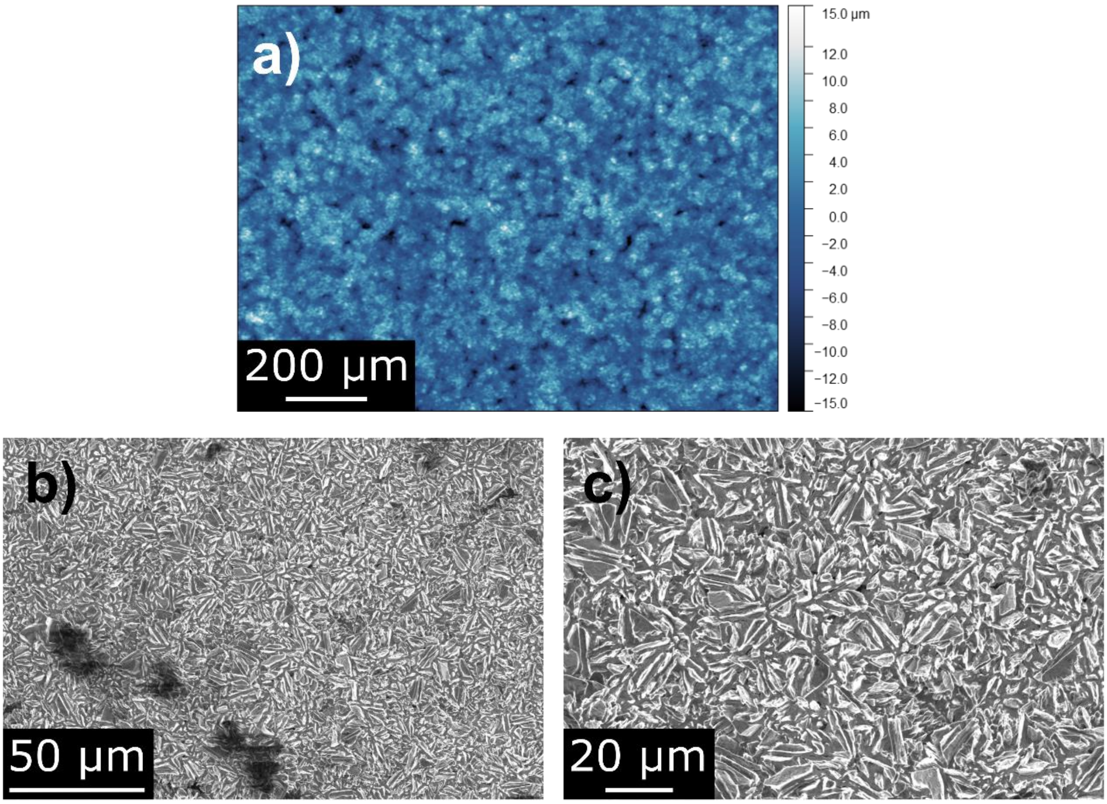

Figure SI.14: Post corrosion characterisation of a thin film BDD anode after 48 hrs electrolysis in 1 M acetic acid in 0.5 M K_2SO_4. a) WLI image of the centre of the corroded region. b) 1000× SEM image of the centre of the corroded region. c) 2000× SEM image of the same region as b.

The S_q from image c is 3.33 μm.

SI.15: Raman Map of Thin Film BDD and EP grade BDD

Raman maps of the EP grade and thin film BDD anodes, pre-corrosion, were collected on a Horiba LabRam HR spectrometer with a 488 nm laser at 100% (nominally 50 mW) power. A 600 l/mm grating was used as well as a ×50LWD objective. The aperture was set to 100 µm in diameter to improve the confocal of the system. Each pixel on the map is an average of two acquisitions, each collected for 1 s. All data was normalised for the number of acquisitions and cosmic rays were automatically supressed by the spectrometer software. All data was baselined and fitted in LabSpec to a gaussian line for the sp^2 carbon feature (centred at ~ 1550 cm^{-1}) and an asymmetric gaussian line for the sp^3 peak (centred at ~1332 cm^{-1}) to consider the line-shape arising from the Fano-resonance due to the high level of boron doping.[1] The maps in Figure SI.15 show the area under these fits, with bright regions having a larger area.

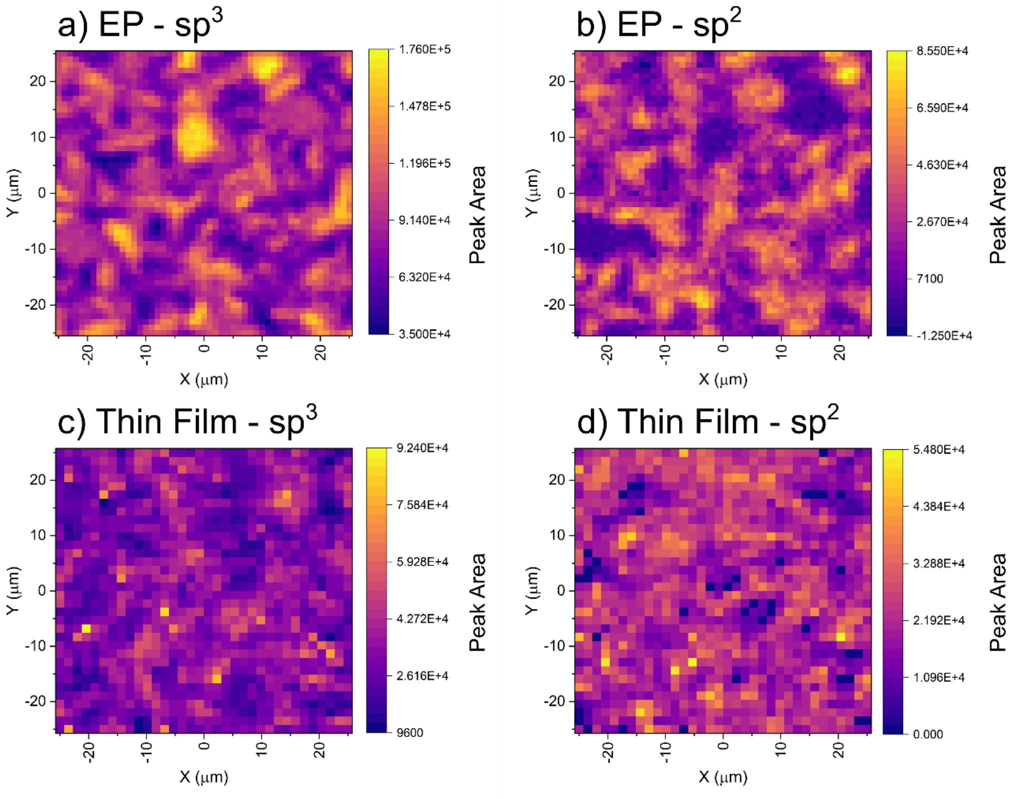

Figure SI.15: Raman maps of the area under the 1332 cm^{-1} peak associated with sp^3 carbon, and the area under the 1550 cm^{-1} peak associated with sp^2 carbon. a) Area under 1332 cm^{-1} sp^3 carbon map for EP grade (freestanding) BDD; b) area under 1550 cm^{-1} sp^2 carbon map for EP grade (freestanding) BDD; c) area under 1332 cm^{-1} sp^3 carbon map for thin film-BDD; d) area under 1550 cm^{-1} sp^2 carbon map for thin film-BDD.

In the case of the sp^3 carbon maps in Figures S1.15a and c as the integrated peak area of the diamond zone centre optical phonon decreases with increasing boron content, the bright areas

likely indicate regions of lower boron doping.[2] For the sp^2 carbon maps in b and d, bright areas indicate regions of high sp^2 carbon. For the EP grade BDD, where the grain size is larger (see Figure 1 main paper), the brightest sp^3 carbon areas (Figure S1.15a) appear to correlate with reduced or negligible sp^2 carbon content (Figure S1.15b). The small grains of the thin film material (see Figure 1 main paper) appear more uniformly doped than the EP grade large grain material, as there is less variation in the sp^3 carbon peak intensity across the sample. The majority of the Raman spectra recorded on the thin film material also shows both sp^3 and sp^2 carbon peaks. This is most likely due to the Raman spot typically interrogating both grains and grain boundaries in a single pixel, given the grain size of the thin film BDD.

Figure SI.16 shows ten representative spectra from each of the maps, the spectra have been normalised so that the diamond Raman line (~ 1332 cm^{-1}) is the same intensity for all. For the thin film sample, all spectra (bar one) show a prominent sp^2 carbon feature at ~ 1550 cm^{-1}. In contrast, for the EP grade BDD half of the spectra show no sp^2 carbon peak.

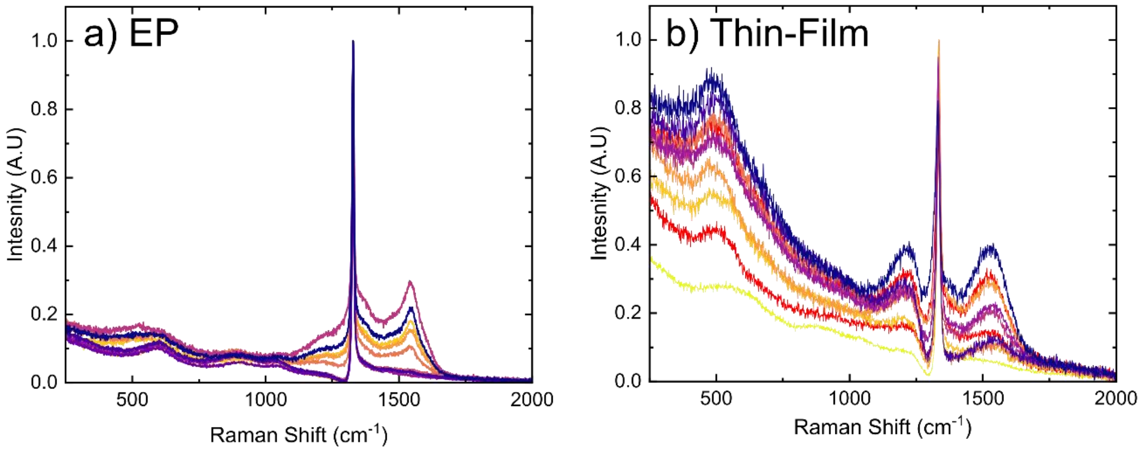

Figure SI.16: c) Ten representative Raman spectra from the EP grade BDD map; d) ten representative Raman spectra from the thin film BDD map. These spectra have been normalised so that the diamond Raman line at ~1332 cm^{-1} is the same intensity for all.

References:

(1) Mortet, V.; Vlčková Živcová, Z.; Taylor, A.; Frank, O.; Hubík, P.; Trémouilles, D.; Jomard, F.; Barjon, J.; Kavan, L. Insight into Boron-Doped Diamond Raman Spectra Characteristic Features. *Carbon N Y* **2017**, *115*, 279–284. https://doi.org/10.1016/J.CARBON.2017.01.022.

(2) Utyuzh, A. N.; Timofeev, Yu. A.; Rakhmanina, A. V. Effect of Boron Impurity on the Raman Spectrum of Synthetic Diamond. *Inorganic Materials* **2004**, *40* (9), 926–931. https://doi.org/10.1023/B:INMA.0000041323.35298.dd.

(3) Hutton, L. A.; Iacobini, J. G.; Bitziou, E.; Channon, R. B.; Newton, M. E.; Macpherson, J. V. Examination of the Factors Affecting the Electrochemical Performance of Oxygen-Terminated Polycrystalline Boron-Doped Diamond Electrodes. *Anal Chem* **2013**, *85* (15), 7230–7240. https://doi.org/10.1021/ac401042t.

(4) Element Six Ltd. *Diafilm^{TM} EP A Solid Solution for Sanitising and Electrochemical Processing*; 2020.
